# Factors associated with diagnosis of stages I and II lung cancer: a multivariate analysis

**DOI:** 10.11606/s1518-8787.2021055003345

**Published:** 2021-12-02

**Authors:** Isabel Cristina Martins Emmerick, Anupama Singh, Maggie Powers, Feiran Lou, Poliana Lin, Mark Maxfield, Karl Uy

**Affiliations:** I University of Massachusetts Medical School Department of Surgery Worcester Massachusetts USA University of Massachusetts Medical School. Division of Thoracic Surgery. Department of Surgery. Worcester, Massachusetts, USA; II University of Massachusetts Medical School Worcester Massachusetts USA University of Massachusetts Medical School. Worcester, Massachusetts, USA

**Keywords:** Lung Neoplasms, diagnosis, Early Detection of Cancer, Survival Analysis, Socioeconomic Factors, Healthcare Disparities

## Abstract

**OBJECTIVE:**

To present the overall survival rate for lung cancer and identify the factors associated with early diagnosis of stage I and II lung cancer.

**METHODS:**

This is a retrospective cohort study including individuals diagnosed with lung cancer, from January 2009 to December 2017, according to the cancer registry at UMass Memorial Medical Center. Five-year overall survival and its associated factors were identified by Kaplan–Meier curves and Cox’s proportional hazards model. Factors associated with diagnosing clinical stage I and II lung cancer were identified by bivariate and multivariate backward stepwise logistic regression (Log-likelihood ratio (LR)) at 95% confidence interval (CI).

**RESULTS:**

The study was conducted with data on 2730 individuals aged 67.9 years on average, 51.5% of whom female, 92.3% white, and 6.6% never smoked. Five-year overall survival was 21%. Individuals diagnosed with early-stage disease had a 43% five-year survival rate compared to 8% for those diagnosed at late stages. Stage at diagnosis was the main factor associated with overall survival [HR = 4.08 (95%CI: 3.62–4.59)]. Factors associated with early diagnosis included patients older than 68 years [OR = 1.23 (95%CI: 1.04–1.45)], of the female gender [OR = 1.47 (95%CI: 1.24–1.73)], white [OR = 1.63 (95%CI: 1.16–2.30)], and never-smokers [OR = 1.37 (95%CI: 1.01–1.86)]; as well as tumors affecting the upper lobe [OR = 1.46 (95%CI: 1.24–1.73)]; adenocarcinoma [OR = 1.43 (95%CI: 1.21–1.69)]; and diagnosis after 2014 [OR = 1.61 (95%CI: 1.37–1.90)].

**CONCLUSIONS:**

Stage at diagnosis was the most decisive predictor for survival. Non-white and male individuals were more likely to be diagnosed at a late stage. Thus, promoting lung cancer early diagnosis by improving access to health care is vital to enhance overall survival for individuals with lung cancer.

## INTRODUCTION

Lung cancer is the leading cause of cancer death worldwide for both men and women. In 2020, lung cancer accounted for the second most common cancer around the world, with 2.21 million cases and 1.80 million deaths^1^. From 2009–2013, deaths due to this cancer in the United States surpassed the records for breast, prostate, colorectal, and liver cancer combined. Moreover, authorities estimate that 131,880 deaths from this disease will occur in the country in 2021^2^.

Stage at diagnosis is the most decisive factor for lung cancer survival. The relative five-year survival rate for localized, stage I, non-small cell lung cancer (NSCLC) is approximately 57.4%, compared to 5% for distant metastases. Surgical resection is the most effective treatment for NSCLC; however, approximately 40% of NSCLC patients are diagnosed at stage 4^3^.

Ethnic disparities exert significant influence on lung cancer diagnosis and treatment. Studies found higher mortality and incidence rates of the disease among African-Americans and non-Hispanics^2,3^. Moreover, Hispanics with stage I lung cancer had lower survival rates when compared with white individuals, besides presenting lower rates of lung resection and higher percentages of late-stage diagnosis^4^. Among other factors, such differences could be explained by the various cultural factors influencing the perception of minoritized groups regarding healthcare, including negative surgical beliefs and mistrust towards the healthcare system and providers^4^.

Individuals with low socioeconomic status and living in resource-deprived areas are at greater disadvantage when seeking timely treatment for lung cancer, which is partly attributed to the time taken to travel to these services. These patients often take longer to attain a proper and timely histological diagnosis and further treatment, which has a negative impact on their survival^7,8^.

For most cancers, early-stage diagnosis is associated with an increased overall survival^9,10^. Although several studies have investigated factors associated with the early-stage diagnosis of cancers affecting oral cavity^10^, breast^9^, and ovary^11^, research on factors associated with lung cancer early diagnosis are still scarce in the literature. Thus, understanding factors associated with early-stage diagnosis is important for identifying possible interventions in the health system and community levels to improve diagnosis and, consequently, survival. We hypothesize that delayed diagnosis and treatment for lung cancer is associated with a series of socioeconomic barriers, thus requiring initiatives to improve the access of minoritized populations and reducing disparities.

This study aims to estimate overall survival for lung cancer and to identify factors associated with diagnosis of clinical stages I and II of lung cancer, including race, socioeconomic status, health insurance status, and education level.

## METHODS

This is a retrospective cohort study conducted with individuals aged 18 years and older who were registered as lung cancer patients at the institutional cancer registry (CR) from January 2009 to December 2017. The study was approved by the Institutional Review Board of the Medical School of University of Massachusetts, under IRB ID: H00008342.

Being part of the University of Massachusetts Cancer Program, accredited by the Commission on Cancer of the American College of Surgeons, the institutional CR was created in 1999. Every year, CR data is directly submitted to the National Cancer Database (NCDB), meeting all NCDB timeliness and data quality criteria. The registry also meets all federal and state requirements, so that incidence rates of all cancer cases diagnosed and/or treated in our medical center are reported to the Massachusetts State Cancer Registry – awarded the North American Association of Central Cancer Registries (NAACCR) Gold Standard for quality, completeness, and timeliness^12^. From this body, cases are reported to the Centers for Disease Control (CDC).

The CR staff collected all the required information on cancer patients through manual record review, following the North American Association of Central Cancer Registries (NAACCR) requirements. Cases are identified by an electronic medical record interface (EPIC interface) that considers information from the pathology department and departments using diagnostic codes from the disease index. This workflow guarantees that the CR is notified of all malignant cases, thus ensuring compliance with federal and state reporting laws. Established by Congress through the Cancer Registries Amendment Act in 1992 and administered by CDC, the National Program of Cancer Registries (NPCR) collects data on cancer occurrence, including type, extent, and location, as well as on the type of initial treatment and outcomes. In the last five years, loss to follow-up was 9.38 %, and the compliance targets for Standard 6.5 was 90%. Every abstractor is subject to a quality review by a third-party company, and CR abstractors scores ranged from 96.78% to 99.52%, which is higher than the required rate of 92%.

Located in the city of Worcester, Central Massachusetts, the health system aims to promote culturally-sensitive excellence in clinical care, service, teaching, and research, thus improving the health of people from diverse communities of Central New England. Despite the high insurance coverage in the state of Massachusetts, disadvantaged patients such as those living in Worcester have more comorbidities and greater need for health education^13^.

This study primary outcome was diagnosis of clinical stage I and II (1, 1A, 1B, 2, 2A, and 2B) lung cancer, whereby the percentage of individuals diagnosed at an early stage was calculated. Clinical staging is based on any information on the extent of the cancer obtained before initiation of thel definitive treatment^14^.

The secondary outcome was overall survival (OS), defined as the length of time from diagnosis (the date the patient was first diagnosed with lung cancer, usually by imaging considered highly suspicious for malignancy or date of biopsy) to death from all causes in months. These outcomes were defined according to the National Cancer Database^14^ and the US Census Bureau^15^.

Missing data accounted for less than 5%, which will reflect on the total number of individuals (N), as shown in Tables 1 and 3.

**Table 1:** Characteristics of lung cancer patients at the UMass Memorial Health Care Center according to the cancer registry from 2009 to 2017.

		n	%
Total		2,730	

Age [Mean (SD)]		67.9	10.9
Age [Median (IQR)]		68.0	60–76
Gender	Female	1,407	51.5
Race	White	2,521	92.3
	Black	61	2.2
	Others	84	3.1
Hispanic origin	Yes	135	4.9
High education level	Yes	1,022	37.4
High income level	Yes	81	3.0
Distance to healthcare service (minutes) [Mean (SD)]		30	66
Distance to healthcare service (miles) [Mean (SD)]		23.9	75.8
Smoking status	Current smoker	1,227	44.9
	Never used	180	6.6
	Previous use	1,281	46.9
Number of comorbidities	0	603	22.1
	1 to 3	860	31.5
	4 to 6	413	15.1
	7 to 10	854	31.3
Health insurance	Medicaid	326	11.9
	Medicare	1,631	59.7
	HMO_PPO	632	23.2
	Non-specified	121	4.4
Histology	adenocarcinoma	1,255	46.0
	squamous cell carcinoma	559	20.5
	Others	851	31.2
Primary site of lesion	Lower Lobe	762	27.9
	Middle Lobe	115	4.2
	Upper Lobe	1,459	53.4
Laterality	Right	1,521	55.7
	Left	1,088	39.9
TNM clinical staging group categories	Stage 1	44	1.6
	Stage 1A	551	20.2
	Stage 1B	137	5.0
	Stage 2, 2A e 2B	170	6.2
	Stage 3	18	0.7
	Stage 3A	285	10.4
	Stage 3B	220	8.1
	Stage 4	1,191	43.6
Year of diagnosis	2014–2017	1,236	45.3
Early-stage diagnosis	Yes (Stage I and II)	902	33.0

**Table 3:** Characteristics of lung cancer patients at the UMass Memorial Health Care by stage, according to the cancer registry from 2009 to 2017.

		Stage I or II		Stage III and IV		Total	
		
		n	%	n	%	n	%
Total		902	34.7	1,696	65.3	2,598	100.0

Age [Mean (SD)]		68.9	10.4	67.6	11.0	68.1	10.8
Age [Median (IQR)]		69.0	62–77	68	60–76	68	60–76
Gender	Female	525	58.2	801	47.2	1,326	51.0
Race	White	851	94.3	1,549	91.3	2,400	92.4
	Black	21	2.3	37	2.2	58	2.2
Hispanic origin	Yes	34	3.8	94	5.5	128	4.9
High education level	Yes	332	36.8	624	36.8	956	36.8
High income level	Yes	32	3.5	42	2.5	74	2.8
Distance to healthcare service (minutes) [Mean(SD)]		30	60	36	1.2	0.6	1.2
Distance to healthcare service (miles)[Mean(SD)]		22.8	68.8	24.6	81.9	24	77.6
Smoking status	Current smoker	356	39.5	818	48.2	1,174	45.2
	Never used	75	8.3	94	5.5	169	6.5
	Previous use	461	51.1	761	44.9	1,222	47.0
Number of comorbidities	No comorbidities	147	16.3	428	25.2	575	22.1
	1 to 3 comorbidities	351	38.9	473	27.9	824	31.7
	4 to 6 comorbidities	137	15.2	249	14.7	386	14.9
	7 to 10 comorbidities	267	29.6	546	32.2	813	31.3
Health insurance	Medicaid	92	10.2	222	13.1	314	12.1
	Medicare	580	64.3	975	57.5	1555	59.9
	HMO_PPO	197	21.8	402	23.7	599	23.1
	Non-specified	30	3.3	83	4.9	113	4.3
Histology	adenocarcinoma	478	53.0	724	42.7	1202	46.3
	squamous cell carcinoma	230	25.5	301	17.7	531	20.4
	Others	194	21.5	671	39.6	865	33.3
Primary site of lesion	Lower Lobe	287	31.8	432	25.5	719	27.7
	Middle Lobe	48	5.3	64	3.8	112	4.3
	Upper Lobe	541	60.0	857	50.5	1398	53.8
Laterality	Right	518	57.4	934	55.1	1452	55.9
	Left	383	42.5	654	38.6	1037	39.9
Year of diagnosis	2014–2017	487	54.0	701	41.3	1188	45.7

Table 1 details other variables used in the study. For determining education and income level, the zip code of patient’s residence reported at the time of diagnosis was used as a proxy. Data on zip codes were collected in the cancer registry and linked with the 2010 census data^15^. The cut-off point was defined as the median values for the state of Massachusetts, so that zip codes referring to median-income geographic locations in the lower bracket characterized patients with income and education levels lower than the state median. Patient’s distance from the healthcare center were calculated using an algorithm was developed using Google Maps.

Survival rates were analyzed considering the (I) adjusted survival analysis (Kaplan-Meier) for the overall cohort; the (II) adjusted survival analysis (Kaplan-Meier) according to stage at diagnosis; and (III) stratified cox proportional hazard models. Survival models were adjusted by age, gender, race, Hispanic origin, education level, income level, distance to healthcare services, smoking status, comorbidities, health insurance, histology, primary site of lesion, laterality, and year of diagnosis.

Factors associated with diagnosis of clinical stage I and II lung cancer (primary outcome) were identified by bivariate and multivariate backward stepwise logistic regression (Log-likelihood ratio statistic (LR)) at 95%CI. Statistical analyses were performed using the Statistical Package for the Social Sciences (SPSS) version 22.

## RESULTS

The cancer registry recorded 2,730 lung cancer patients from January 2009 to December 2017. All patients were followed until August 31^st^, 2018, and five were lost to follow-up. Most patients were female (51.5%) and white (94.6%), with average age of 67.9 years and median age of 68.0 years (IQR 60–76); 6.6% of them never smoked. Among the study cohort, 3% lived in areas with income level higher than the state median and 37% in areas with education level higher the state median (Table 1).

In general, lung cancer patients presented a high number of comorbidities, with only 22.1% of them showing no comorbid conditions. Most patients (69%) were diagnosed at a late stage, either clinical stages 3 or 4 (Table 1).

The five-year overall survival for the study cohort was 21%, approaching 43% for patients diagnosed with early-stage lung cancer (Stage I and II) and dropping to 8% among individuals diagnosed at more advanced stages (Stage III and IV) (Figure). The median survival rate was a little over one year (12.7 months) for the overall cohort, reaching 49 months for diagnosed at an early stage and dropping to 9 months among those diagnosed at late stages.

**Figure:**
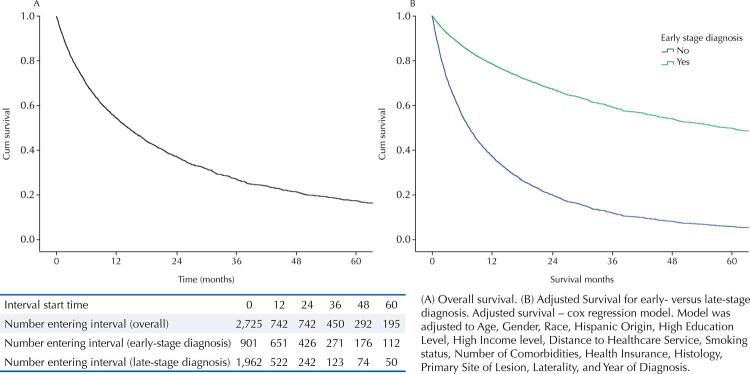
Five-year adjusted survival curves: (A) overall survival and (B) survival by stage at diagnosis

Late-stage diagnosis was the most decisive factor for lung cancer overall survival [HR = 4.08 (95%CI: 3.62–4.59)]. The final model included being older than 68 [(HR = 1.64; 95%CI: 1.49–1.80)] and male [(HR = 1.15 (95%Cl: 1.05–1.27)]; having smoking habits [HR = 1.35 (95%Cl: 1.11–1.63)], more than three comorbidities [(HR = 1.37 (95%Cl: 1.25–1.50)], histological diagnosis other than adenocarcinoma [HR = 1.27 (95%Cl: 1.15–1.39)], lower or middle lobe as primary site of lesion [HR = 1.10 (95%Cl: 1.00–1.21)]; and being diagnosed before 2014 [HR = 1.24 (95%Cl: 1.12–1.37)] (Table 2). We found neither race nor ethnicity to be associated with overall survival. Similarly, survival was not associated with education level and mildly associated with living in a neighborhood with income level lower than the state median [HR = 1.30 (95%Cl: 0.99–1.72)], *p*-value = 0.071 in the bivariate analysis); however, such association was not maintained in the final cox regression model.

**Table 2:** Factors associated with overall survival in the cox proportional hazard final model, UMass Memorial Health Care cancer registry from 2009 to 2017.

	HR	95%CI		p
Diagnosed at late stage vs. early stage	4.08	3.62	4.59	0.000
Older than 68	1.64	1.49	1.80	0.000
Male	1.15	1.05	1.27	0.004
Current or previous smoker	1.35	1.11	1.63	0.002
More than 3 comorbidities	1.37	1.25	1.50	0.000
Non-adenocarcinoma	1.27	1.15	1.39	0.000
Lower or middle lobe as primary site of lesion	1.10	1.00	1.21	0.046
Diagnosed before 2014	1.24	1.12	1.37	0.000

As stage at diagnosis was the most significant variable for a greater survival rate, we investigated factors associated with a higher likelihood of being diagnosed at stage I and II disease. Among the 2,598 cancer patients, 902 (35%) were diagnosed with early-stage lung cancer (Table 3). This percentage increased over the years, going from 19.3% in 2009 to 39.9% in 2017.

According to the bivariate analysis, early diagnosis was associated with: age greater than 68 years [OR = 1.25 (95%CI: 1.06–1.47)]; female gender [OR = 1.46 (95%CI: 1.32–1.83)]; white [OR = 1.58 (95%CI: 1.14–2.20)]; never-smokers [OR = 1.40 (95%CI: 1.05–1.89)]; upper lobe as primary site of lesion [OR = 1.47 (95%CI: 1.25–1.73)]; adenocarcinoma [OR = 1.51 (95%CI: 1.29–1.78)]; diagnosis after 2014 [OR = 1.67 (95%CI: 1.42–1.96)]; and health insurance (Medicare or Medicaid) [OR = 1.22 (95%CI: 1.02–1.46)]. We found no association between early diagnosis and income or education level, nor with distance to healthcare services.

The most important factor associated with early diagnosis in the final multivariate analysis was being white [OR = 1.63 (95%CI: 1.16–2.30)], followed by year of diagnosis [OR = 1.61 (95%CI: 1.37–1.90)]. Age greater than 68 years [OR = 1.23 (95%CI: 1.04–1.45)], female gender [OR = 1.47 (95%CI: 1.24–1.73)], never-smokers [OR = 1.37 (95%CI: 1.01–1.86)], upper lobe as primary site of lesion [OR = 1.46 (95%CI: 1.24–1.73)], and adenocarcinoma [OR = 1.43 (95%CI: 1.21–1.69)] were other relevant factors associated with the outcome (Table 4).

**Table 4:** Factors associated with diagnosis of clinical stages I and II lung cancer in the final multivariate logistic regression, UMass Memorial Health Care cancer registry from 2009 to 2017.

	OR	95%CI		p
Age (older than 68 years old)	1.23	1.04	1.45	0.01
Female	1.47	1.24	1.73	0.00
White	1.63	1.16	2.30	0.00
Never-smoker	1.37	1.01	1.86	0.04
Upper lobe as primary site of lesion	1.46	1.24	1.73	0.00
Adenocarcinoma	1.43	1.21	1.69	0.00
Diagnosis after 2014	1.61	1.37	1.90	0.00

## DISCUSSION

Our results indicate that stage at diagnosis was the primary factor associated with lung cancer survival. Even after adjusting for other variables, diagnosis at stages I and II was associated with a higher survival rate when compared to late-stage diagnosis (III and IV). Corroborating the literature on the theme^16,17^, we found worse survival rates among older individuals. Moreover, male individuals showed poor overall survival when compared to females, also in line with other studies findings^18,19^. Individuals who never smoked presented increased survival at all stages diagnosis, which reiterates the fact that heavy smokers often have an unhealthy lifestyle, worsening their overall survival^20^.

We verified an association between increased survival of lung cancer patients and diagnosis after 2014, which is coherent with the advent of new therapies and techniques^21^. Stage at diagnosis and treatment are more important predictors of survival than race, suggesting that racial disparities in lung cancer survival may disappear provided that early detection efforts benefit both Black and white individuals in the same way^22^. This can be verified, for example, by the reduction in racial disparities in timely cancer treatment arising from the expansion of the Medicaid insurance health coverage^23^.

Over the last 10 years, lung cancer incidence rate has been decreasing at a 2.3% rate yearly, while that of death decreased at approximately 2.9%^3^. The percentage of early-stage diagnosis is increasing over time, which might indicate a progress on lung cancer awareness and a reflection of the compliance with screening recommendations. However, different populations still face great disparities in diagnosis and treatment^23^, suggesting a multifactorial problem. The ethnic and racial differences surrounding cancer care are complex, extending beyond access to healthcare – a broad term that includes not only the means to visit medical providers, but also the possibility of doing so timely. Thus, cancer care includes both individual components as well as health policies regulating care^7^.

Identifying factors associated with early-stage diagnosis allow us to define intervention points within the health system that could improve access and quality of care, thus increasing overall survival.

In this study, we verified considerable race and gender disparities in early-stage lung cancer diagnosis. Several factors associated with early diagnosis – female gender, older age, and white race – have likewise been correlated with greater access to care and increased survival in other studies^26^. Besides racial disparities reflected on the finding that Black patients are often diagnosed with lung cancer at later stages, these individuals also have lower stage-specific survival for most cancer types. When compared to white patients, the relative risk of death among Black individuals is 33% higher, even after adjusting for gender, age, and stage at diagnosis^2^.

The gender differences in healthcare are multifactorial and influenced by structural, psychosocial, and behavioral determinants of health^19,22,23^. Studies show that women tend to seek more healthcare regarding mental and physical issues than men^30^, which might explain why female individuals accounted for a greater percentage of patients diagnosed with early-stage lung cancer in our study.

An important aspect of this study is that the cohort does not reflect the environment where the institution is located. Located in a diverse country, Worcester is likewise a relatively diverse city, whose population consists of 57.1% white individuals, 20.9% Hispanic or Latinos, 11.8% African-American, and 7.29% Asians^24^. However, our study cohort consisted of 94.6% white individuals, 2.3% African-Americans, and 3.2% other races. White individuals are almost twice as likely to be diagnosed with lunger cancer at an early stage. A recent study showed that Medicaid expansion as part of the Affordable Care Act (ACA) reduced racial disparities in access to care^23^. This finding indicates that strategies, policies, and programs must use the health system as an instrument to reduce structural disparities surrounding proper and timely access to healthcare.

This study did not intended to capture all predictors of access to diagnosis, for our cohort consists of individuals that have already accessed the healthcare system. Thus, further studies should adopt a community level approach to determine the barriers of access to healthcare.

This study has some limitations as to the census variables used to estimate income and education level, given that the zip code served as reference for analysis. For example, ‘High-income’ refers to areas whose median income was greater than the median income of the city of Massachusetts. However, individuals median income may be higher than that of the zip code in which they resides, so that their access to healthcare services may be different than the overall access of the population from that particular zip code area.

Despite being a single-center study, our study findings echo those from the literature, providing evidence of the important disparities in lung cancer diagnosis and treatment and highlighting the need for addressing to provide a more equitable access to health.

## CONCLUSIONS

Stage at diagnosis was the most decisive factor for lung cancer survival. Non-white and male individuals were more likely of being diagnosed at late stages. This study providing information for the population attended at this institution, besides outlining the possible pathways to reduce inequities.

In a continuous effort to improve early diagnosis and equitable access to healthcare, further studies are needed to identify the barriers to access to lung cancer diagnosis and treatment at the community level, thus helping to reduce mortality and enhance overall survival.
